# Association between omega-3 polyunsaturated fatty acids and osteoarthritis: results from the NHANES 2003–2016 and Mendelian randomization study

**DOI:** 10.1186/s12944-024-02139-4

**Published:** 2024-05-17

**Authors:** Yuxuan Liu, Feichao Song, Muchun Liu, Xi Huang, Shuyan Xue, Xuanyu Zhang, Huiqin Hao, Junfeng Zhang

**Affiliations:** 1 School of Management, Shanxi Medical University, Jinzhong, China; 2 Academy of Medical Sciences, Shanxi Medical University, Taiyuan, China; 3 Third Clinical College, Shanxi University of Traditional Chinese Medicine, Jinzhong, China; 4 School of Basic Medicine, Shanxi University of Traditional Chinese Medicine, Jinzhong, China

**Keywords:** Osteoarthritis, Omega-3 fatty acids, Mendelian randomization, National health and nutrition examination survey

## Abstract

**Background:**

Omega-3 polyunsaturated fatty acids (omega-3 PUFAs) exhibit potential as therapeutics for a variety of diseases. This observational and Mendelian randomization (MR) study aims to explore the relationship between omega-3 PUFAs and osteoarthritis (OA).

**Methods:**

Excluding individuals under 20 years old and those with missing data on relevant variables in the National Health and Nutrition Examination Survey (NHANES) spanning from 2003 to 2016, a total of 22 834 participants were included in this cross-sectional study. Weighted multivariable-adjusted logistic regression was used to estimate the association between omega-3 PUFAs and OA in adults. Moreover, restricted cubic splines were utilized to examine the dose-response relationship between omega-3 PUFAs and OA. To further investigate the potential causal relationship between omega-3 PUFAs and OA risk, a two-sample MR study was conducted. Furthermore, the robustness of the findings was assessed using various methods.

**Results:**

Omega-3 PUFAs intake were inversely associated with OA in adults aged 40 ∼ 59 after multivariable adjustment $[\text{OR} (95\% \text{CI): }0.85 (0.73, 0.98), P = 0.027]$, with a nonlinear relationship observed between omega-3 PUFAs intake and OA $\left(P \text{ for non-linearity}\text{ = 0.034}\right)$. The IVW results showed there was no evidence to suggest a causal relationship between omega-3 PUFAs and OA risk $\text{[OR} (95\% \text{CI): }0.967 (0.863, 1.084), P = 0.568]$.

**Conclusions:**

Omega-3 PUFAs were inversely associated with OA in adults aged 40 ∼ 59. However, MR studies did not confirm a causal relationship between the two.

**Supplementary Information:**

The online version contains supplementary material available at 10.1186/s12944-024-02139-4.

## Introduction

Osteoarthritis (OA) is a complex joint disease. If severe, it may lead to joint dysfunction, deformities, and disability, significantly impacting patient’s quality of life and well-being [1]. There are approximately 595 million patients worldwide [2, 3]. OA stands as a primary contributor to disability among elderly individuals, particularly those aged 60 and older [3, 4]. Nowadays, the burden of OA continues to increase, posing a major public health challenge and threat. Consequently, to develop scientific prevention strategies and reduce the disease burden of OA, further investigation of potential risk factors for OA is required.

Nutritional and dietary habits play a crucial role in preventing or treating OA [5]. Numerous studies have demonstrated omega-3 Polyunsaturated fatty acids (PUFAs)’ nutritional importance in chronic illnesses owing to their antioxidant and anti-inflammatory features [6, 7]. In arthritic rats, omega-3 PUFAs have exhibited anti-inflammatory effects [8]. In vitro studies suggest that specific omega-3 PUFAs, particularly eicosapentaenoic acid (EPA), may inhibit cartilage deterioration in patients with chronic inflammatory joint disorders [9]. Kuszewski’s study found that fish oil (docosahexaenoic acid (DHA): 2 000 mg/d + EPA: 400 mg/day) has the potential to alleviate pain associated with OA in overweight elderly individuals [10]. Similarly, a meta-analysis reported that supplementation with omega-3 PUFAs can help alleviate pain and increasing joint function [11]. However, in a study on an American elderly population, researchers confirmed that omega-3 PUFAs supplementation failed to alleviate knee joint pain [12]. These inconsistencies across studies underscore the need for further research to clarify the relationship between omega-3 PUFAs and OA.

Based on the existing evidence, this study hypothesized that changes in omega-3 PUFAs intake might affect OA. This is the first study to integrate observational and MR studies to investigate the association between omega-3 PUFAs and OA.

## Methods

### Observational study

#### Study population

The National Health and Nutrition Examination Survey (NHANES) began in 1960 and continuous data collection began in 1999, with a 2-year cycle for public release [13, 14]. This program operates as an ongoing program using a cross-sectional, population-based design [13]. NHANES collects information on population health, lifestyle, and disease risk through questionnaires, laboratory tests, and physical examinations.

**Fig. 1:**
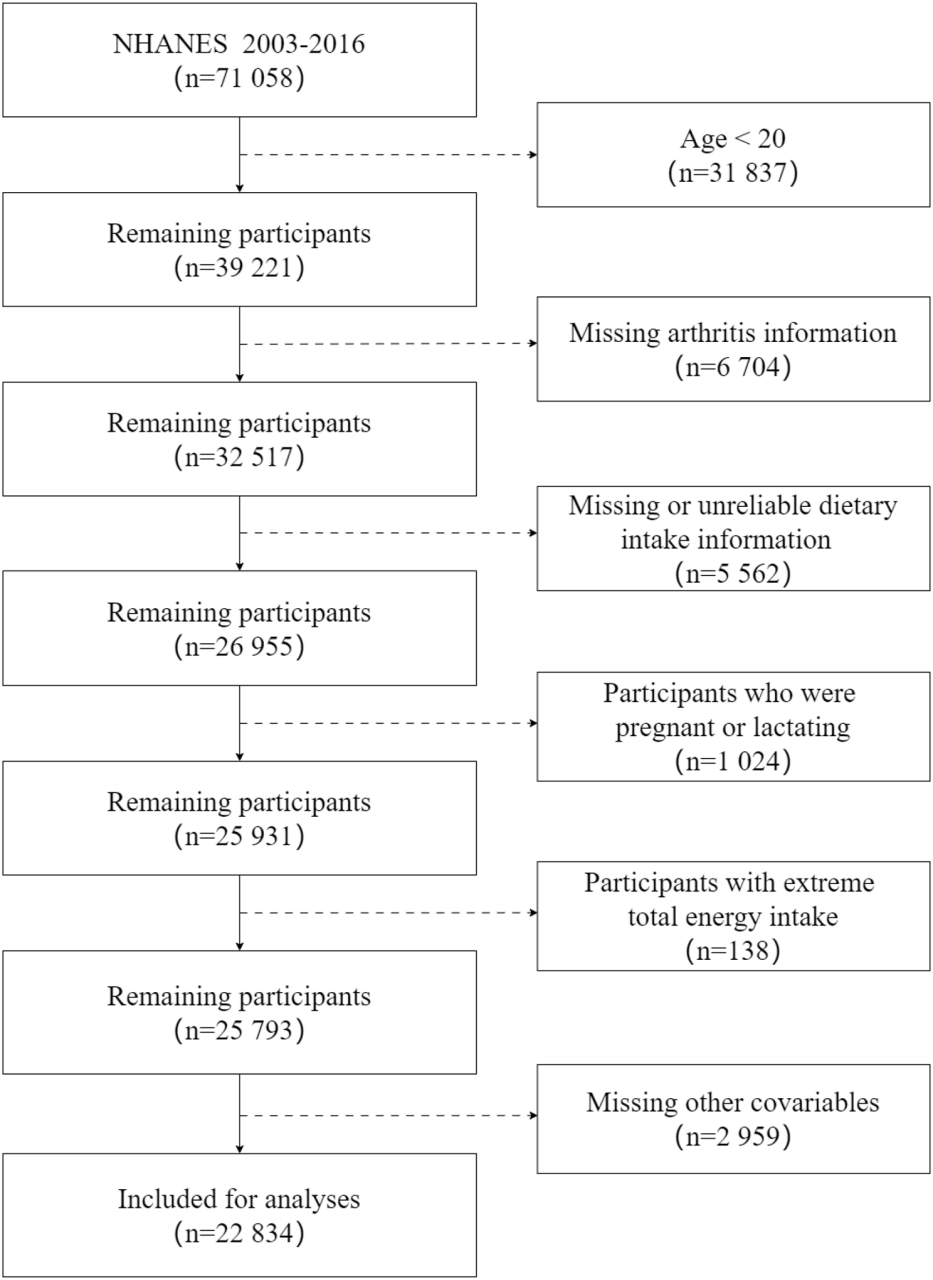
NHANES 2003–2016 sample selection flowchart. Abbreviations: NHANES: National Health and Nutrition Examination Survey

For this study, seven cycles were selected and combined (2003–2004, 2005–2006, 2007–2008, 2009–2010, 2011–2012, 2013–2014, 2015–2016) with a total of 71 058 participants. Participants younger than 20 years old (*n* = 31 837), with missing data on arthritis (*n* = 6 704), missing or unreliable data on the 24-hour dietary recalls (*n* = 5 562), pregnancy or breastfeeding (*n* = 1 024), extreme total energy intake (< 500 kcal/day or > 8 000 kcal/day for males and < 500 kcal/day or > 5 000 kcal/day for females) (*n* = 138), and missing other covariables (*n* = 2 959) were excluded, 22 834 participants were ultimately included (Fig. 1).

#### Dietary omega-3 PUFAs intake and osteoarthritis

NHANES uses an automated multiple-pass method in five steps to quantify and assess 24-hour dietary intake from individuals [15]. The first-day dietary intake was obtained at the Mobile Examination Center, and the second-day dietary intake was recorded during a scheduled telephone interview 3∼10 days later.

The total omega-3 PUFAs included DHA$\left(\text{22: 6n-3}\right)$, DPA $\left(\text{22: 5n-3}\right)$, EPA $\left(\text{20: 5n-3}\right)$, SDA $\left(\text{18: 4n-3}\right)$, and ALA $\left(\text{18: 3n-3}\right)$. Omega-3 PUFA intake from the two 24-hour dietary recalls was averaged during the analysis. If there were only data from the first day, this value was used as the analysis data instead of the average.

To assess OA, the participants were asked two questions about arthritis. First, “Has a doctor ever told you that you have arthritis?” Participants who replied “no” to this inquiry were considered to be non-osteoarthritis (non-OA) individuals, while those who responded “yes” were subsequently questioned on “What type of arthritis?” Participants who provided the response “Osteoarthritis or degenerative arthritis” were considered to be OA patients.

#### Covariables of interest

①Sociodemographic characteristics: age, gender, race, level of education, and poverty-to-income ratio (PIR) were collected during household interviews. ②Anthropometric measurements: Body Mass Index (BMI) (kg/m^2^). ③Lifestyle characteristics: drinking and smoking status were collected through questionnaires, and based on previously published literature, participants were grouped [16, 17]. Total energy intake was obtained from 24-hour dietary recalls. ④Health condition: cardiovascular disease (CVD), diabetes and hypertension (yes or no). The above information is all from the participants’ self-report.

#### Statistical analysis

The sampling design used by NHANES is extremely complex, which allows the selected sample to represent the U.S. population [18]. Because seven NHANES survey cycles were selected, a new sampling weight was reconstructed, allowing all statistical analyses to be adjusted based on the survey design and weight variables to restore the sampling structure of the NHANES.

Demographic characteristics, lifestyle characteristics, anthropometric measurements, health condition and dietary intake were described statistically for the total sample, as well as for OA and non-OA individuals.

Firstly, this study investigated the association between omega-3 intake and OA when omega-3 are continuous variables. Secondly, omega-3 intake was further categorized into quartiles, and using Q1 as a reference. Three multivariable logistic regression models were formulated. Model 1 was a crude model without adjusting for any variables. Model 2 was adjusted for age. Building upon Model 2, Model 3 incorporated sex, race, level of education, PIR, smoking, drinking, BMI (kg/m^2^), total energy intake, CVD, diabetes and hypertension (yes or no). In addition, their dose-response relationships were estimated using restricted cubic splines (RCS) with 4 knots. Data processing and analyses were conducted using R 4.2.3.

### Mendelian randomization studies

#### Data source of omega-3 PUFAs and OA

A Genome-Wide Association Studies (GWAS) meta-analysis of circulating metabolites in 13 544 Europeans identified single nucleotide polymorphisms (SNPs) linked with plasma omega-3 PUFAs [19]. Summary data for OA were derived from the United Kingdom Biobank (UKB) for 63 556 European samples, including 12 658 self-reported patients with OA and 50 898 controls [20] (Table 1). All study participants were of European ancestry, which reduced the population stratification bias to some extent.

**Table 1:** Information on data sources in the MR study

Exposure/ Outcome	GWAS ID	Sample size (cases)	Number of SNPs	Population	Data sources	Year of publication
Omega-3 PUFAs	met-c-855	13 544	11 401 623	European	GWAS meta-analysis	2016
OA	ebi-a-GCST005811	63 556	15 870 475	European	UK biobank	2018

*Abbreviation* MR: Mendelian randomization; PUFAs: Polyunsaturated fatty acids; OA: Osteoarthritis; GWAS: Genome-Wide-Association Studies; SNPs: Single nucleotide polymorphisms

#### SNPs selection and assumption

**Fig. 2:**
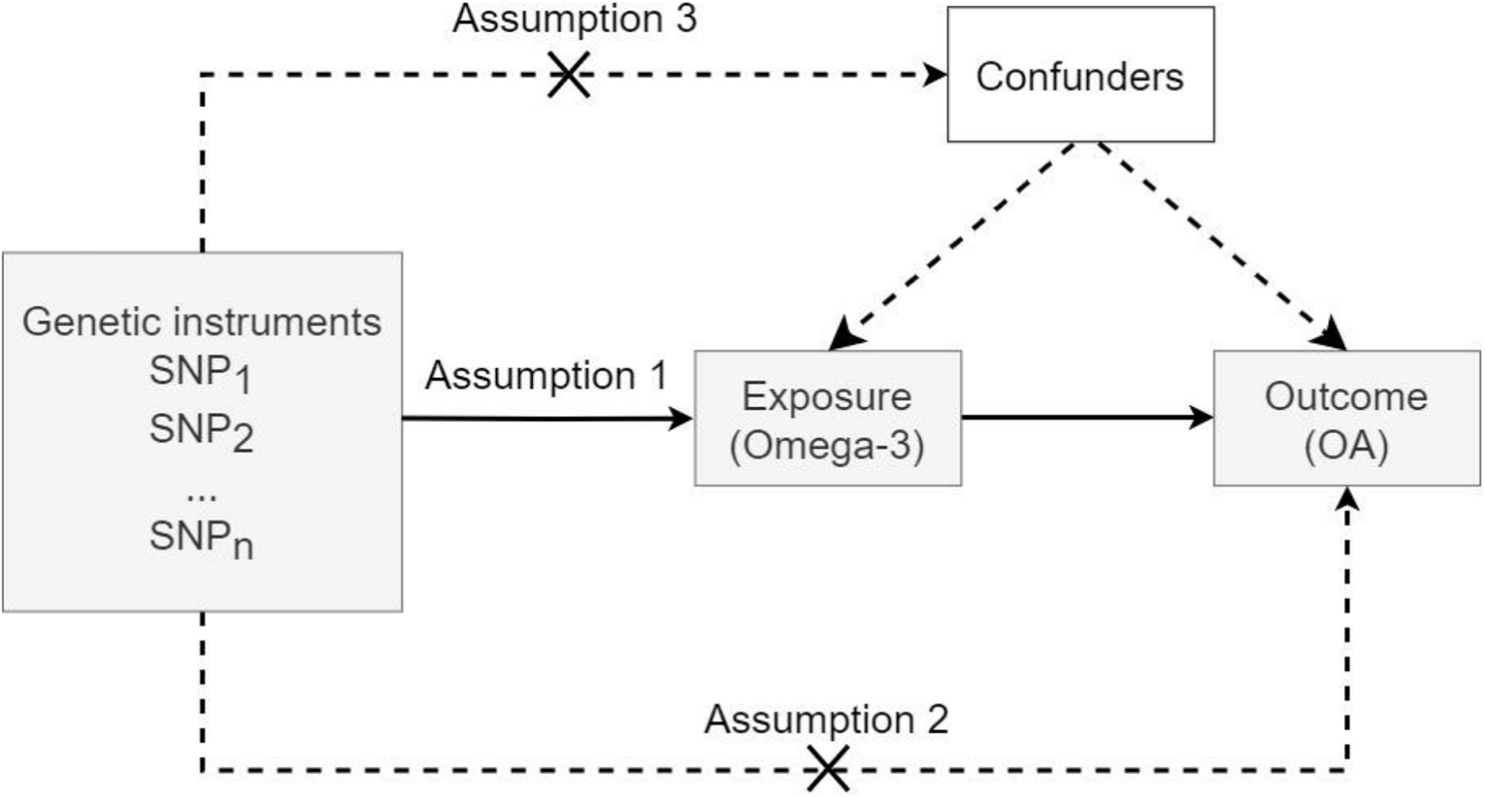
Diagram of Mendelian randomization study design. *Abbreviation* OA: Osteoarthritis; SNP: Single nucleotide polymorphism

As shown in Fig. 2, to ensure the robustness of the two-sample Mendelian randomization (MR) study, the following three core assumptions were strictly adhered to: ① selected SNPs need to be highly associated with exposure; ② selected SNPs are not directly related to the outcome; ③ selected SNPs cannot be associated with other confounders that may affect exposure or outcome. According to the STROBE-MR guidelines [21, 22], the process of selecting instrumental variables was as follows: ① in order to select SNPs that meet Assumption 1, a statistically significant threshold $\left( P < 5 \times 10^{-8} \right)$was established; because of the limited number of significant SNPs selected under this standard, the standard was relaxed to $\left( P < 5 \times 10^{-6} \right)$. ② Performed linkage disequilibrium (LD) test: $\text{kb=10 000}$ and ${\text{r}}^{\text{2}}\text{ <0.001}$. ③ Excluded 6 SNPs related to potential confounders such as BMI and weights by searching the PhenoScanner V2 [23] and LDtrait database [24]. ④ Calculate the F-statistic for each SNP, identifying weak instrumental factors as $F\text{ < 10}$. The following formula is for calculating the F- statistic of a single instrumental variable [25]:

$$\eqalign{ F = {{{R^2}\left( {N - 2} \right)} \over {1 - {R^2}}}; & \cr & {R^2} = \left[ {2 \times {\beta ^2} \times eaf \times \left( {1 - eaf} \right)} \right]\,\,/ \cr & \,[2 \times {\beta ^2} \times eaf \times \left( {1 - eaf} \right)\, \cr & + \,(2 \times N \times SE{\left( \beta \right)^2}\, \times \,eaf \times (1 - eaf))] \cr}$$

where ${R}^{2}$ represents the degree of exposure (omega-3) explained by instrumental variables (IVs), N is the sample size of GWAS for exposure(omega3), $\beta$ is the estimated genetic effect on exposure (omega-3), eaf is the effect allele frequency, SE$\left(\beta \right)$ is the standard error of $\beta$ [25]. Since the weak instrumental variables violate Assumption 1, they must be eliminated if they exist. In addition, the MR-Pleiotropy RESidual Sum and Outlier (MR-PRESSO) is used to test for outliers. If there are outliers, they will be removed before conducting MR analysis. Twelve eligible SNPs were finally screened for inclusion in this study (Table S1).

#### Statistical analysis

The major approach for MR analysis was the inverse variance-weighted (IVW) method (random effects), which combines wald estimates for each SNPs to obtain an overall estimate, thus providing a relatively accurate causal assessment when all SNPs satisfy MR assumptions [26–28]. The weighted median (WM), MR-Egger method, MR-PRESSO, and MR Robust adjusted profile score (MR-RAPS) were used as additional analytical approaches. To ensure the quality of the findings, the heterogeneity and pleiotropy test, and the leave-one-out test were conducted. The heterogeneity test included Cochrane’s Q test and MR-PRESSO global test; the directional pleiotropy test included the MR Egger’s intercept test, and all the above analyses needed to be observed to test the *P* value. If $P \text{ > 0.05}$, it indicates that there is no enough evidence to show heterogeneity or pleiotropy. The leave-one-out test calculates the combined effect of the remaining SNPs by excluding a single SNP, in order to assess whether any single SNP has a remarkable impact on the causal association. Data processing and analysis were conducted using the TwoSampleMR 0.5.8 software package in R 4.2.3.

## Results

### Observational study

This research involved 22 834 participants, including 2 831 patients with OA. A comprehensive description of the characteristics of the entire sample, as well as those with or without OA, is presented in Table 2. The average age of the sample was 46 years, with 51% males and 49% females. Elderly women are more likely to develop OA. Patients with OA had higher BMI than non-OA individuals.

**Table 2:** Participant baseline characteristics from NHANES 2003–2006

Characteristic	*N*	Overall, *N* = 22 834 (100%)	Non-OA, *N* = 20 003 (87%)	OA, *N* = 2 831 (13%)	*P* Value
**Age**	22 834	46 (17)	43 (16)	61 (13)	**< 0.001**
**Sex**	22 834				**< 0.001**
Male		11 888 (51%)	10 852 (53%)	1 036 (35%)	
Female		10 946 (49%)	9 151 (47%)	1 795 (65%)	
**Race**	22 834				**< 0.001**
Mexican American		3 727 (8.3%)	3 501 (9.2%)	226 (2.8%)	
Other Hispanic		1 907 (4.8%)	1 742 (5.2%)	165 (2.1%)	
Non-Hispanic White		10 588 (70%)	8 686 (67%)	1 902 (85%)	
Non-Hispanic Black		4 572 (11%)	4 168 (11%)	404 (5.9%)	
Other Race - Including Multi-Racial		2 040 (6.8%)	1 906 (7.1%)	134 (4.2%)	
**Educational level**	22 834				> 0.9
Less Than 9th Grade		2 069 (4.4%)	1 840 (4.5%)	229 (4.2%)	
9 ∼ 11th Grade		3 073 (9.9%)	2 709 (10%)	364 (9.9%)	
High School Grad/GED		5 212 (22%)	4 558 (22%)	654 (22%)	
Some College or AA degree		6 832 (32%)	5 962 (32%)	870 (33%)	
College Graduate or above		5 648 (31%)	4 934 (31%)	714 (31%)	
**Poverty-income ratio**	22 834	3.06 (1.64)	3.05 (1.65)	3.19 (1.60)	**0.006**
**Smoke**	22 834				**< 0.001**
Never smoker		12 622 (55%)	11 313 (56%)	1 309 (46%)	
Former smoker		5 362 (24%)	4 298 (22%)	1 064 (38%)	
Current smoker		4 850 (21%)	4 392 (22%)	458 (16%)	
**Alcohol**	22 834				**< 0.001**
Non-drinker		6 141 (22%)	5 260 (22%)	881 (26%)	
1 ∼ 5 drinks/month		11 323 (49%)	9 981 (50%)	1 342 (48%)	
5 ∼ 10 drinks/month		1 941 (10%)	1 794 (11%)	147 (6.5%)	
10 + drinks/month		3 429 (18%)	2 968 (18%)	461 (19%)	
**BMI(kg/m²)**	22 834	29 (7)	28 (6)	31 (7)	**< 0.001**
**Total omega-3 (g/day)**	22 834	1.76 (0.93)	1.77 (0.93)	1.70 (0.92)	**0.001**
**Energy (kcal/day)**	22 834	2 147 (818)	2 180 (831)	1 931 (687)	**< 0.001**
**Diabetes (Yes)**	22 834	2 399 (7.7%)	1 818 (6.4%)	581 (16%)	**< 0.001**
**Hypertension (Yes)**	22 834	1 977 (6.9%)	1 349 (5.2%)	628 (18%)	**< 0.001**
**Cardiovascular disease (Yes)**	22 834	7 164 (28%)	5 464 (24%)	1 700 (55%)	**< 0.001**

*Abbreviation* OA, Osteoarthritis; Non-OA, non-Osteoarthritis; BMI, Body mass index

**Fig. 3:**
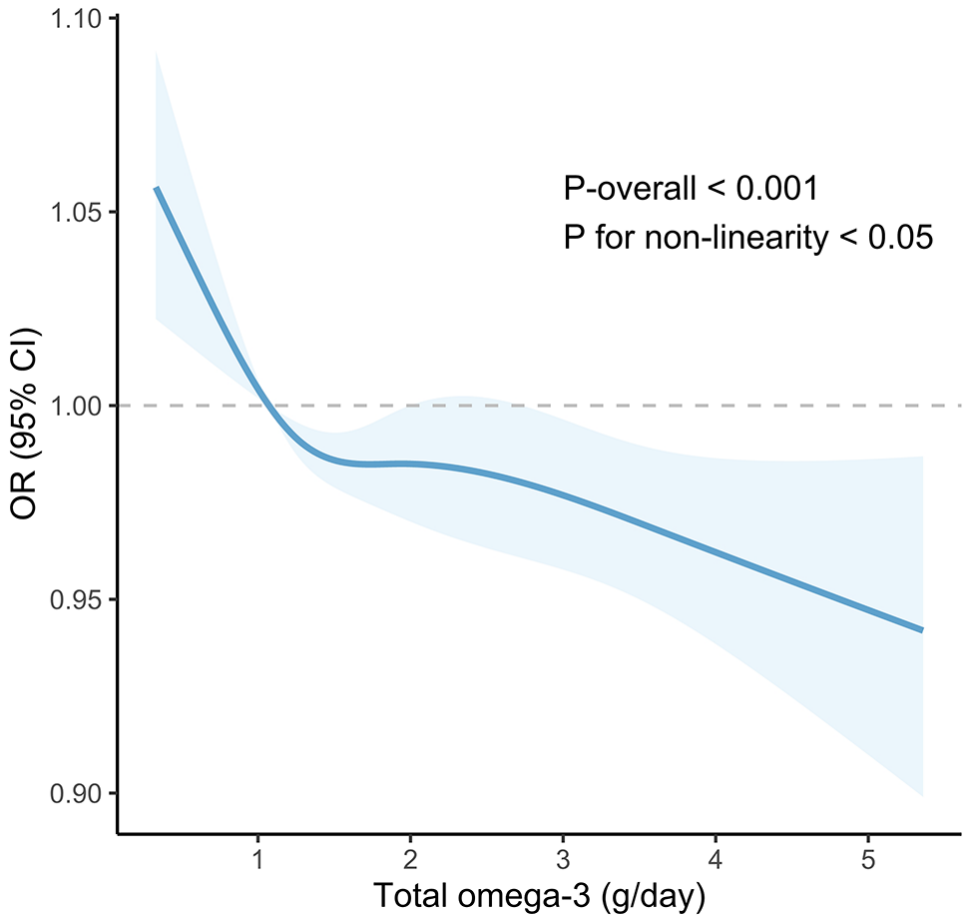
RCS curves of omega-3 PUFAs intake and OA dose-response. Abbreviations: RCS: Restricted cubic splines; PUFAs: Polyunsaturated fatty acids; OA: Osteoarthritis; OR: Odd ratio; CI: Confidence interval

Compared to those who consumed a low amount of omega-3 PUFAs, individuals who consumed a larger amount of omega-3 PUFAs had a reduced prevalence of OA $\text{[}\text{OR }(95\% \text{CI}\text{): 0.91 (0.86, 0.97), }P \text{< 0.001],}$ according to the univariate analyses (Table 3). However, this relationship disappeared after adjusting for covariables in Model 2 and Model 3. In subgroups stratified by age, the negative correlation between omega 3 and OA is significant in adults aged 40 ∼ 59 years old$\text{[}\text{OR }(95\% \text{CI}\text{): 0.86 (0.77, 0.96), }P \text{< 0.001]}$. The association remained statistically significant in the multivariable logistic regression Model 2 and Model 3 (Table 4). These findings implied that the population aged 40 ∼ 59 who intake omega-3 PUFAs could help prevent OA. In addition, the RCS curves showed a nonlinear correlation between omega-3 PUFAs and OA in adults aged 40 ∼ 59 (Model 1: $P\text{ for non-linearity }\text{= 0.033}$; Model 2: $P\text{ for non-linearity }\text{= 0.028}$; Model 3: $P\text{ for non-linearity}\text{ = 0.034}$) (Fig. 3).

**Table 3:** Weighted logistic regression analysis results of omega-3 intake and OA

	Model 1^1^ OR (95% CI)	Model 2^2^ OR (95% CI)	Model 3^3^ OR (95% CI)
Total omega-3 (g/day)	0.91 (0.86, 0.97) ^**^	0.98 (0.91, 1.04)	0.96 (0.88, 1.05)
Total omega-3 (g/day, quartile)			
Q1	Reference	Reference	Reference
Q2	0.94 (0.81, 1.08)	1.03 (0.88, 1.22)	1.00 (0.84, 1.18)
Q3	0.82 (0.72, 0.93) ^**^	0.95 (0.81, 1.12)	0.95 (0.79, 1.14)
Q4	0.79 (0.68, 0.91) ^**^	0.94 (0.79, 1.11)	0.88 (0.71, 1.09)

^1^Model 1: adjusted for none

^2^Model 2: adjusted for age (years)

^3^Model 3: adjusted for age (years), sex, race, PIR, smoking, drinking, BMI, energy (kcal/day), CVD, diabetes, hypertension

^*^P < 0.05; ^**^P < 0.01

*Abbreviation:* BMI: Body mass index; PIR: Family income-to-poverty ratio; CVD: Cardiovascular disease; OR: Odds ratio; CI: Confidence interval

**Table 4:** Age stratified weighted logistic regression analysis results of omega-3 intake and OA

	Model 1^1^ OR (95% CI)	Model 2^2^ OR (95% CI)	Model 3^3^ OR (95% CI)
Total omega-3 (g/day)			
20 ∼ 39	0.98 (0.81, 1.20)	0.96 (0.79, 1.18)	0.91 (0.70, 1.18)
40 ∼ 59	0.86 (0.77, 0.96) ^**^	0.84 (0.75, 0.94) ^**^	0.85 (0.73, 0.98) ^*^
≥ 60	1.05 (0.97, 1.14)	1.07 (0.98, 1.16)	1.07 (0.97, 1.18)

*Abbreviation* BMI: Body mass index; PIR: Family CVD: Cardiovascular disease; OR: Odds ratio; CI: Confidence interval

^*^P < 0.05; ^**^P < 0.01

^1^Model 1: adjusted for none

^2^Model 2: adjusted for age (years)

^3^Model 3: adjusted for age (years), sex, race, PIR, smoking, drinking, BMI, energy (kcal/d), CVD, diabetes, hypertension

### Mendelian randomization studies

**Fig. 4:**
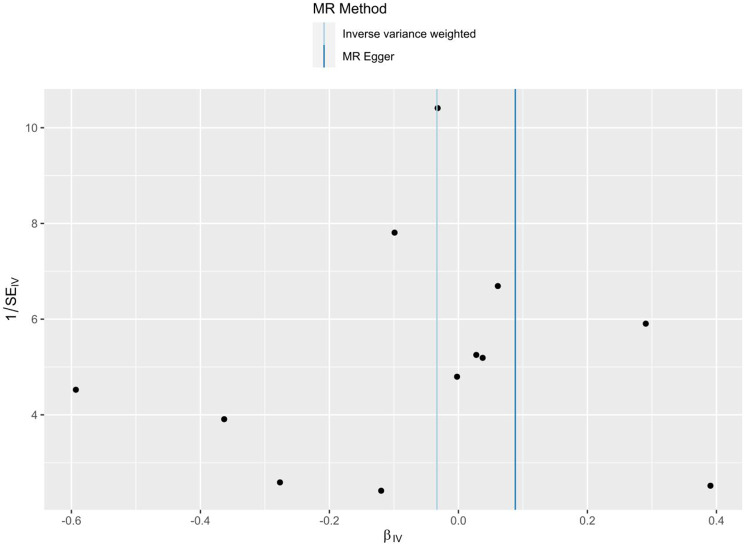
Funnel plot of MR results. MR: Mendelian randomization

**Fig. 5:**
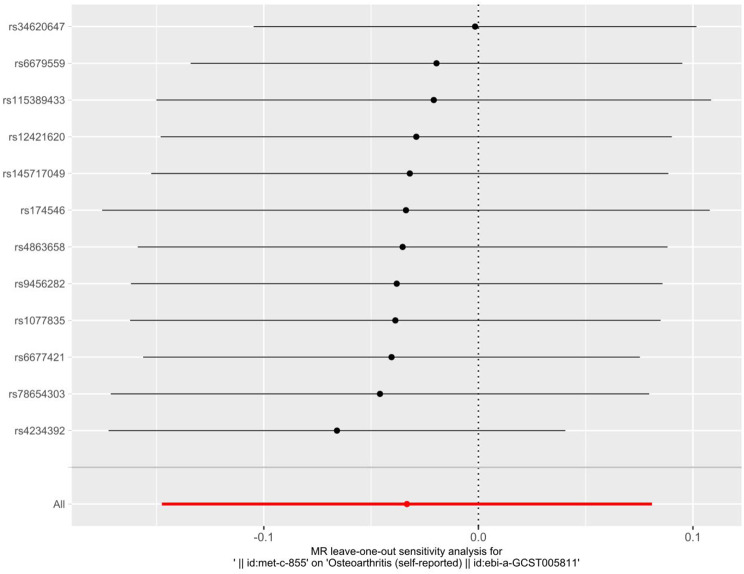
Analytical diagram of the leave-one-out method

As shown in Table 5, the IVW findings revealed that there was no causal association between omega-3 PUFAs and OA $\text{[}\text{OR }(95\% \text{CI}\text{): }\text{0.967}\text{ (}\text{0.86}\text{3}\text{, }\text{1.084}\text{), }P \text{= 0.5}\text{68}\text{]}$. Other methods further confirmed that there was no evidence to suggest a causal relationship between the two. The results of MR-PRESSO global test, MR-Egger intercept analysis, and Cochrane’s Q test did not show significant evidence of pleiotropy or heterogeneity (Table 6). The funnel plot showed a symmetrical distribution of causal effects (Fig. 4). The leave-one-out test indicated that no SNPs had a remarkable impact on the estimation of causal relationship (Fig. 5). These results indicate that the MR analysis results were robust.

**Table 5:** Mendelian randomization estimates for omega-3 PUFAs and OA

Exposure	Outcome	Methods	OR (95%CI)	*P*
Omega-3 PUFAs	OA	MR Egger	1.092 (0.864, 1.380)	0.477
		Weighted median	0.981 (0.855, 1.126)	0.786
		Inverse variance weighted	0.967 (0.863, 1.084)	0.568
		MR-PRESSO	0.967 (0.863, 1.084)	0.579
		MR-RAPS	0.966 (0.871, 1.071)	0.512

*Abbreviation* OR, Odd ratio; MR-PRESSO, MR-Pleiotropy RESidual Sum and Outlier; MR-RAPS, MR Robust adjusted profile score

**Table 6:** The results of MR-PRESSO, MR-Egger intercept, and Cochrane’s Q test

Exposure	Outcome	MR-PRESSO global test		MR-Egger intercept analysis			Cochran Q test		
		Outliers	*P*	Intercept	se	*P*	Q value	Q_df	*P*
Omega-3 PUFAs	OA	No outlier	0.267	0.002	0.017	0.896	14.240	11	0.220

*Abbreviation* MR-PRESSO, Mendelian Randomization Pleiotropy RESidual Sum and Outlier

## Discussion

Omega-3 PUFAs, as essential fatty acids for health, the human body cannot produce them; therefore, they must be ingested through food. Prior research has demonstrated that consuming omega-3 PUFAs has positive effects on OA. A meta-analysis showed that patients with OA experienced excellent pain relief and improved joint function with omega-3 PUFAs supplementation [11]. Recently, a 6-month randomized controlled trial showed that daily intake of krill oil supplements can moderately improve knee joint pain and stiffness [29]. Although the specific mechanism of omega-3’s protective effect on OA is not yet clear, most studies have been conducted from the perspective of anti-inflammatory features of omega-3. Omega-3 PUFAs may have an inhibitory effect on the expression of inflammatory markers like $\text{interleukin}\text{-}\text{1}\beta$; in previous research, the effects of omega-3 PUFAs enriched green-lipped mussels (GLM) on pain and joint degradation caused by OA were investigated in experimental rats with OA. The results demonstrated that after administration of GLM, the production of $\text{IL}\text{-}\text{1}\beta$ and $\text{IL}\text{-}\text{6}$ significant decreased [30]. However, a previous 26-week clinical study showed no significant change in Western and McMaster University Osteoarthritis Index scores in the glucosamine sulfate plus omega-3 group compared to the glucosamine sulfate alone [31]. Similarly, another study found that compared to the placebo group, the group receiving cod liver oil supplements did not improve pain caused by OA better [32]. As there is no consensus on the association between omega-3 PUFAs and OA, this study utilized a large open-access database to assess the link between the two.

This study consisted of two parts: first, the observational study found that omega-3 is independently associated with OA. But subgroup analysis showed that omega-3 PUFAs might be inversely associated with OA in individuals aged 40 ∼ 59 years. Regardless of whether the model was employed to adjust for covariables, the aforementioned findings persisted. Although the observational study adjusted for a wide range of potential confounders, it was not possible to completely adjust all confounding factors. Moreover, due to the inherent limitations of the observational study, causal inference cannot be made [33–35]. Therefore, a two-sample MR study was conducted as a supplementary approach for causal inference. However, the results of MR study did not support a causal relationship between omega-3 and OA risk.

There is limited research exploring the association between omega-3 and OA in a large and representative population, and the research results are also inconsistent [36, 37]. The cross-sectional study indicated that omega-3 is independently associated with OA. Interestingly, a negative association between omega-3 and OA was found in the subgroup aged 40 ∼ 59 years, while no such association was observed in other age groups. Differences in the pathogenesis and influencing factors of OA in different age groups may be the reason for the lack of association in other age groups. OA, as a degenerative aging disease of joints, tends to occur in middle-aged and elderly people. With the increase of age, the physiological functions of the human body gradually degenerate, and the metabolic rate slows down, which makes the elderly more vulnerable to various diseases. Prevalent risk factors such as obesity, metabolic syndrome, and diabetes, common among older adults, could amplify OA risk and potentially obscure or counterbalance the association between omega-3 and OA.

The results of the MR study did not support a causal association between omega-3 and OA risk, and this result has been validated using various MR analysis methods. Furthermore, there was no evidence to suggest heterogeneity or pleiotropy. This result is robust. Although potential confounding factors were adjusted for as much as possible in the NHANES analysis, there are still many potential covariables that were not comprehensively considered, and due to data limitations, many of the data, including omega-3 intake, were based on participants’ self-reports, which may lead to bias. This may be the reason for the inconsistency between the results of MR studies and observational studies, and this is also the significance of the MR study. Furthermore, it should be noted that MR analysis estimates the effect of exposure over a lifetime, rather than at specific time points. Therefore, it is not yet known whether increasing omega-3 can prevent OA at specific times in life, such as in a certain age group.

Additionally, it is also necessary to emphasize one issue. All studies conducted with omega-3 have shown that the beneficial effects are primarily reflected in the improvement of pain and stiffness, while almost no impact on the structural progress of the disease has been observed [38]. Therefore, the role of omega-3 may be more reflected in treatment rather than prevention.

### Study strengths and limitations

This study has several strengths: first, the large amount of data in the NHANES database allowed us to obtain a sufficiently large sample size and high-quality data. Second, all analyses were weighted such that the sample was a better representation of the U.S. population as a whole. Third, the nonlinear relationship between omega-3 PUFAs and OA was explored by constructing an RCS. The greatest strength of this study is the combination of observational research and MR analysis. This is the first study to integrate observational and MR data to evaluate the association between omega-3 PUFAs and OA. It is difficult to draw causal conclusions from observational studies; therefore, MR analysis can compensate for this limitation. Moreover, MR studies use publicly available GWAS data for causal inference with a larger sample size and higher statistical strength.

However, it is unavoidable that this research has a few limitations, including the following: first, omega-3 intake was derived from dietary recalls of participants, potentially introducing recall bias. Second, there may be potential confounding factors that have not been adjusted for, such as medication data that could potentially influence OA. Third, this study only includes Americans and Europeans and lacks data from other populations, resulting in limited extrapolation. Therefore, more data from other populations are required for a comparative analysis. Furthermore, in the MR study, first, due to the limited sample size of GWAS for omega-3, the significant threshold was relaxed to $\left( P < 5 \times 10^{-6} \right)$ to increase the number of available IVs, potentially leading to weak instruments bias. Second, MR studies only examined linear causal relationships between omega-3 and OA, but the observational studies indicate that there is a non-linear relationship between the two in the age group of 40 ∼ 59 years old, so the non-linear causal relationship needs to be tested. What’s more, this was only a fundamental theoretical study, and more animal experiments and cohort studies are needed to confirm these conclusions for better clinical application.

## Conclusion

The results of the MR analysis of this study indicated that there was no evidence of a causal association between omega-3 and OA, although the observational study showed an inverse association between omega-3 intake and OA in adults aged 40 ∼ 59. These results provide novel insights for directing therapeutic decision-making. The dietary therapies that target the composition of the diet to enhance the consumption of foods rich in omega-3 PUFAs need to be carefully considered. Its therapeutic potential for the prevention or mitigation of OA needs to be further evaluated, and it is possible that dietary therapies may only be beneficial for a subset of the population. For example, it may require to be administered at a particular age. Hence, the results need to be further verified by large-scale, high-quality longitudinal trials or randomized controlled trials, and investigation into the potential mechanism between them needs to be further explored.

### Electronic supplementary material

Below is the link to the electronic supplementary material.

Supplementary Material 1

## Data Availability

The data used in observational and MR studies can be publicly obtained from NHANES (https://www.cdc.gov/nchs/nhanes) and OpenGWAS (https://gwas.mrcieu.ac.uk/).
