# Supplementary material for: Association between omega-3 polyunsaturated fatty acids and osteoarthritis: results from the NHANES 2003–2016 and Mendelian randomization study
Source: Lipids Health Dis. 2024 May 17;23:147. doi: 10.1186/s12944-024-02139-4 (PMC11100232; doi:10.1186/s12944-024-02139-4)
Supplement: Supplementary file 1 — Supplementary Material 1 [file 12944_2024_2139_MOESM1_ESM.docx]

Table S1: Genetic instruments for omega-3 and their associations with OA.

| SNPs | EA | OA | Exposure (Omega-3 FA) | | | |  | Outcome (OA) | | |
| --- | --- | --- | --- | --- | --- | --- | --- | --- | --- | --- |
|  |  |  | Beta | SE | *P* | F |  | Beta | SE | *P* |
| rs10051260^a^ | A | G | 0.072 | 0.015 | 1.59E-06 | 23.347 |  | -0.008 | 0.018 | 0.678 |
| rs1077835 | G | A | 0.089 | 0.014 | 1.08E-09 | 37.694 |  | 0.003 | 0.017 | 0.845 |
| rs112875651^a^ | A | G | -0.066 | 0.013 | 5.16E-07 | 25.549 |  | -0.007 | 0.014 | 0.625 |
| rs115389433 | T | A | -0.463 | 0.100 | 4.35E-06 | 21.392 |  | 0.046 | 0.059 | 0.442 |
| rs11604424^a^ | T | C | -0.090 | 0.014 | 3.32E-10 | 40.026 |  | 0.033 | 0.018 | 0.061 |
| rs12421620 | A | G | -0.146 | 0.028 | 1.91E-07 | 27.496 |  | 0.040 | 0.056 | 0.476 |
| rs1260326^a^ | C | T | -0.097 | 0.013 | 3.37E-14 | 58.303 |  | 0.019 | 0.014 | 0.189 |
| rs12886210^b^ | C | T | -0.059 | 0.012 | 1.93E-06 | 22.978 |  | -0.040 | 0.014 | 0.005 |
| rs143988316^a^ | T | C | -0.171 | 0.024 | 2.95E-12 | 49.397 |  | 0.009 | 0.026 | 0.733 |
| rs145717049 | T | C | -0.191 | 0.033 | 6.67E-09 | 34.095 |  | 0.023 | 0.079 | 0.773 |
| rs174546 | T | C | -0.154 | 0.012 | 1.19E-34 | 152.776 |  | 0.005 | 0.015 | 0.738 |
| rs34620647 | C | A | 0.094 | 0.019 | 5.33E-07 | 25.487 |  | -0.056 | 0.021 | 0.007 |
| rs4234392 | T | A | -0.257 | 0.054 | 2.03E-06 | 22.878 |  | -0.075 | 0.043 | 0.089 |
| rs4863658 | C | T | 0.075 | 0.014 | 2.35E-07 | 27.089 |  | 0.000 | 0.016 | 0.992 |
| rs6677421 | A | G | 0.166 | 0.035 | 3.07E-06 | 22.072 |  | 0.065 | 0.066 | 0.329 |
| rs6679559 | T | C | 0.059 | 0.013 | 4.84E-06 | 21.182 |  | -0.021 | 0.015 | 0.155 |
| rs7224790^a^ | C | G | 0.176 | 0.034 | 3.28E-07 | 26.437 |  | -0.010 | 0.034 | 0.762 |
| rs78654303 | A | C | 0.209 | 0.043 | 1.59E-06 | 23.352 |  | 0.013 | 0.031 | 0.683 |
| rs9456282 | G | A | 0.088 | 0.018 | 7.94E-07 | 24.707 |  | 0.002 | 0.017 | 0.884 |

^a^ rs10051260, rs112875651, rs11604424, rs1260326, rs143988316, and rs7224790 was associated with Body Mass Index or Weight and was therefore excluded. ^b^rs12886210 was the outlier that was removed before MR analysis. SNPs, single nucleotide polymorphisms; EA, effect allele; OA, other allele; EAF, effect allele frequency; SE, standard error.
